# Two Putative Cypovirus-Encoded miRNAs Co-regulate the Host Gene of GTP-Binding Nuclear Protein Ran and Facilitate Virus Replication

**DOI:** 10.3389/fphys.2021.663482

**Published:** 2021-08-04

**Authors:** Su Lin, Yongsheng Wang, Ze Zhao, Wanming Wu, Yun Su, Zhendong Zhang, Manman Shen, Ping Wu, Heying Qian, Xijie Guo

**Affiliations:** ^1^School of Biotechnology, Jiangsu University of Science and Technology, Zhenjiang, China; ^2^Sericultural Research Institute, Chinese Academy of Agricultural Sciences, Zhenjiang, China

**Keywords:** silkworm, cypovirus, microRNA, target gene, *BmRan*, virus replication

## Abstract

microRNA (miRNA) plays important roles in regulating various biological processes, including host-pathogen interaction. Recent studies have demonstrated that virus-encoded miRNAs can manipulate host gene expression to ensure viral effective multiplication. *Bombyx mori* cypovirus (BmCPV), a double-stranded RNA virus with a segmented genome, is one of the important pathogens for the economically important insect silkworm. Our present study indicated that two putative miRNAs encoded by BmCPV could promote viral replication by inhibiting the gene expression of *B. mori* GTP-binding nuclear protein Ran (*BmRan*), an essential component of the exportin-5-mediated nucleocytoplasmic transport of small RNAs. BmCPV-miR-1 and BmCPV-miR-3 are two of the BmCPV-encoded miRNAs identified in our previous studies. *BmRan* is a common target gene of them with binding sites all located in the 3′-untranslated region (3′-UTR) of its mRNA. The expression levels of the two miRNAs in the midgut of larvae infected with BmCPV gradually increased with the advance of infection, while the expression of the target gene *BmRan* decreased gradually. The miRNAs and the recombinant target gene consisting of reporter gene *mCherry* and 3′-UTR of *BmRan* mRNA were expressed in HEK293T cells for validating the interaction between the miRNAs and the target gene. qRT-PCR results revealed that BmCPV-miR-1 and BmCPV-miR-3 negatively regulate target gene expression not only separately but also cooperatively by binding to the 3′-UTR of *BmRan* mRNA. By transfecting miRNA mimics into BmN cells and injecting the mimics into the body of silkworm larvae, it was indicated that both BmCPV-miR-1 and BmCPV-miR-3 could repress the expression of *BmRan* in BmN cells and in the silkworm, and the cooperative action of the two miRNAs could enhance the repression of *BmRan* expression. Furthermore, the repression of *BmRan* could facilitate the replication of BmCPV genomic RNAs. It is speculated that BmCPV-miR-1 and BmCPV-miR-3 might reduce the generation of host miRNAs by inhibiting expression of *BmRan*, thus creating a favorable intracellular environment for virus replication. Our results are helpful to better understand the pathogenic mechanism of BmCPV to the silkworm, and provide insights into one of the evasion strategies used by viruses to counter the host defense for their effective multiplication.

## Introduction

*Bombyx mori* cypovirus (BmCPV) is one of the important pathogens of the silkworm (Cao et al., 2012). It is a typical RNA virus belonging to the *Cypovirus* genus of *Reoviridae*. Its genome consists of 10 segmented double-stranded RNAs with a total length of more than 24 kbp, each encoding a structural or non-structural protein (Hagiwara et al., 2002; Hu et al., 2019). The virus infects only midgut epithelial cells in the silkworm and produces polyhedra in the cytoplasm. The intact virus particles are generally embedded in the polyhedra and must be released in the larval midgut to infect the silkworm. The infection of the virus to the silkworm always causes serious cytoplasmic polyhedrosis disease resulting in big losses to the commercial sericultural production. The mechanism of interaction between the virus and silkworm needs to be further explored for the development of effective strategies to control the occurrence and prevalence of the disease. In the silkworm, major antiviral defense mechanisms such as RNA interference (RNAi), NF-kB-mediated, Imd (immune deficiency), stimulator of interferon gene (STING), and Janus kinase/signal transducer and activator of transcription (JAK/STAT) pathways have been shown to play important roles in antiviral immunity (Jiang, 2021). In contrast, viruses can modulate prophenol oxidase (PPO), phosphatidylinositol 3-kinase (PI3K)/protein kinase B (Akt), and extracellular signal-regulated kinase (ERK) signaling pathways of the host to elevate their proliferation in the silkworm (Jiang, 2021). Transcriptome studies of silkworm have revealed a complex response against BmCPV infection. While, studies of deep sequencing of viral small RNAs have indicated the importance of the RNAi pathway in the control of cypovirus infection although many functional aspects still need to be elucidated and conclusive evidence is lacking (Swevers et al., 2020).

microRNA (miRNA) is a type of 19–25 nt single-stranded non-coding small RNAs, which is widely found in animals, plants, and nematodes, and regulates the expression of target genes at the post-transcriptional level (Filipowicz et al., 2008). A miRNA can target and regulate multiple target genes, and a target gene may also be regulated by multiple miRNAs. At the same time, studies have shown that the increase in the number of miRNA binding sites in mRNA 3′-untranslated region (3′-UTR) can enhance the translational repression of a target gene (Heiss et al., 2012). Similar studies also showed that co-operativity between two or more miRNA-binding sites enhanced repression of mRNA translation via an unknown mechanism when sites were separated by 13–35 nucleotides (Saetrom et al., 2007). Many viruses also encode miRNAs and 569 miRNAs of virus origin have been registered in the miRBase database version 22.1 (2018)^1^. Virus-encoded miRNAs play an important role in the intricate interaction between virus and hosts, including regulation of host immune response, evasion from recognition by the host immune system (Liang et al., 2014), inhibition of apoptosis (Zhao et al., 2011), regulation of cell cycle (Gottwein et al., 2007), mimicking host miRNAs (Grimson et al., 2007; Zhao et al., 2009), and so on. At present, most viral miRNAs reported are encoded by DNA viruses, but some RNA viruses can also encode functional miRNAs (Swaminathan et al., 2013; Qiu et al., 2018). Our previous deep sequencing of small RNA in the midgut of silkworm larvae infected by BmCPV virus identified some virus-derived non-coding RNA sequences similar to miRNA. Further study proved that a BmCPV-encoded miRNA can regulate the expression of host genes and affect the replication and proliferation of the virus (Guo et al., 2020).

The present work studies the functions of two putative BmCPV-encoded miRNAs, namely BmCPV-miR-1 and BmCPV-miR-3. Target gene prediction against the silkworm genome identified that the *B. mori* GTP-binding nuclear protein ran gene (*BmRan*) is the common target gene of the two miRNAs. Their regulation on the target gene and its influence on BmCPV virus replication were analyzed. Studying the BmCPV-encoded functional miRNAs and revealing their functions in the process of pathogen-host interaction would enrich the miRNA family encoded by viruses and help to reveal the miRNA-mediated new mechanism of regulation on RNA virus replication and proliferation.

## Materials and Methods

### Silkworm Strain, Cell Lines, and Virus

The domesticated silkworm of strain 4008 used in this study was supplied by Silkworm Germplasm Conservation Center, Chinese Academy of Agricultural Sciences. BmCPV, BmN cell, and HEK293T cell (human embryonic kidney cells) lines were maintained in our laboratory. miRNA mimics, inhibitors, and negative controls (NCs) were synthesized and chemically modified by Shanghai GenePharma Co., Ltd. The pmCherry-N1 plasmid, lentiviral expression vector pLNHX, pLKO.3G, and packaging plasmids pVSV-G, pSPAX2, and pMD2.G were purchased from Wuhan MiaoLingBio Inc. and kept in our laboratory.

### Virus Inoculation and Tissue Collection

*Bombyx mori* cypovirus polyhedra suspension at a concentration of 1.0 × 10^8^ mL^–1^ was coated on fresh mulberry leaves cut into 5 × 3 cm. The leaves coated with the virus suspension were fed to the 5th instar silkworm larvae and the average amount of virus ingested by each larva was calculated to be about 1.0 × 10^6^ polyhedra. Another group of larvae fed with mulberry leaves coated with sterile water were used as blank control. When the mulberry leaves coated with virus or water were all eaten up (about 6 h), the larvae were given fresh mulberry leaves and reared under the standard condition of 14 h light and 10 h darkness and relative humidity of about 90%.

The larvae were dissected at 12, 24, 48, 72, and 96 h, respectively after inoculation to collect midguts. The collected midgut was rinsed in DEPC water to remove the attached mulberry leaf pieces and put into a cryotube after the extra water was absorbed with tissue paper. The midguts of every five larvae were mixed as one sample and three samples were taken at each time point. Then the samples were stored at −80°C after quick freezing in liquid nitrogen.

### RNA Extraction

In this study, the expression level of both the virus-derived miRNAs and the host target gene, and the replication level of viral genome in the midgut of the silkworm larvae needed to be quantitatively detected, respectively. Therefore, total RNA was extracted with a classic manual method of the following steps. The frozen silkworm larval midguts were ground into fine powder with liquid nitrogen, put into an RNase free centrifuge tube. A total of 1 mL of lysate RL was added into the tube, and the mixture was shaken and mixed, then incubated at room temperature for 5 min. It was centrifuged at 4°C, 12000 rpm for 15 min. Then the supernatant was mixed with 200 μL of chloroform in a new tube, shaken sufficiently, and kept at room temperature for 10 min, followed by centrifugation at 4°C, 12000 rpm for 15 min. The upper aqueous phase was mixed with an equal volume of pre-cooled isopropanol in a new RNase free centrifuge tube, kept at 4°C for 10 min, then centrifuged at 4°C, 12000 rpm for 10 min. The supernatant was discarded, the pellet was washed with 1 mL of 75% ethanol and centrifuged at 4°C, 7500 rpm for 5 min. Then, the supernatant was discarded and the pellet was kept at room temperature for 5–10 min to allow the RNA pellet to dry. The RNA precipitate was dissolved with 30–50 μL of RNase-free water, and stored at −80°C after the concentration was measured.

### cDNA Synthesis

cDNA was synthesized with a TaKaRa Primer Script^TM^ RT reagent kit for reverse transcription (Takara Biomedical Technology Co., Ltd., China) according to the manufacturer’s instructions. For the first step, 2 μL of 5 × gDNA Eraser Buffer, 1 μL of gDNA Eraser, and 1 μg of RNA were mixed in the reaction tube to a total volume of 10 μL by adding ddH_2_O, then kept at 42°C for reaction for 2 min. Then, 10 μL of the reaction solution, 4 μL of 5 × Prime Script Buffer 2, 1 μL of Prime Script RT Enzyme Mix I, 1 μL of RT Primer, and 4 μL of ddH_2_O were mixed, and the reaction was performed with the program 37°C, 15 min, 85°C, 5 s, and 4°C + ∞. After the reaction, the synthesized cDNA was stored at −20°C. The cDNA for miRNA detection was synthesized with stem-loop RT primers. The stem-loop primers were designed with reference to literature (Chen et al., 2005) and the sequences are shown in Table 1.

**TABLE 1:** Primer sequences for qRT-PCR.

Primer name	Sequence (5′–3′)
**Stem-loop reverse-transcribed PCR for BmCPV-miR-1**	
	**RT:** GTCGTATCCAGTGCAGGGTCCGAGGT ATTCGCACTGGATACGACTAGTGT
	**F:** ACACTCCAGCTGGGGAAATGGACACAGGC
**Stem-loop reverse-transcribed PCR for BmCPV-miR-3**	
	**RT:** GTCGTATCCAGTGCAGGGTCCGAGGTATTCGCA CTGGATACGACATCAAGCC
	**F:** ACACTCCAGCTGGGTAGGAGAATTAGCGCGG
Universal	**R:** CCAGTGCAGGGTCCGAGGTA
mCherry	**F:** CTCAGTTCATGTACGGCTCCAAGG
	**R:** GGAGTCCTGGGTCACGGTCAC
Human β-actin	**F:** CTCCATCCTGGCCTCGCTGT
	**R:** GCTGTCACCTTCACCGTTCC
*BmRan*	**F:** GCCGTAACGACTTTGCTTTGGAAC
	**R:** TTGCCAGTACCACCATCTCCTACC
Genomic RNA S2	**F:** GTTGAGCGTCAGCAGTCAGATCG
	**R:** TGTTTACCCTGAGCAGCGTTATCG
Genomic RNA S5	**F:** CGCTTACAGGCAGTGGAATAGGAC
	**R:** GCTCTAACACATCGCTGGGCTAAG
Genomic RNA S10	**F:** ACCGTCAGTGATTGCTCGTGTAAC
	**R:** AGCGTCACCCTATCCGAAGACC
Bmβ-actin	**F:** CCGTATGAGAAAGGAAATCA
	**R:** TTGGAAGGTAGAGAGGGAGG

### qRT-PCR

Primer Premier 5.0 software was used to design quantitative primers (Table 1) for target gene *BmRan*, reporter gene *mCherry*, and internal reference gene β-*actin* (Table 1), and the stem-loop primers for miRNA quantitative detection were designed with reference to literature (Chen et al., 2005). The reaction system was prepared according to the instructions of the SYBR premix Ex Taq^TM^ kit (Takara Biomedical Technology Co., Ltd., China) (2 × SYBR Premix Ex Taq^TM^: 10 μL, ROX Reference Dye: 0.4 μL, upstream primer: 0.8 μL, downstream primer: 0.8 μL, cDNA: 1 μL, ddH_2_O: 7 μL, total volume: 20 μL). Three technical replicates were set for each quantitative reaction and the reaction was run on an ABI Prism fluorescence quantitative PCR instrument (Applied Biosystems, Foster City, CA, United States). The reaction program was 95°C for 45 s followed by 45 cycles of 95°C for 5 s, and 60°C for 31 s. *Bm*β-*actin* and *BmTIF-4A* were used as internal reference genes for detection of target gene expression. The differences in gene expression levels were calculated using the relative quantitative 2^–ΔΔCT^ method (Chang et al., 2009).

### Target Gene Prediction

The target genes of BmCPV-miR-1 and BmCPV-miR-3 was predicted against the silkworm genome by the miRanda and Targetscan^2^ software. The genes that could be predicted by both the two software programs and that mainly participated in immune response, escape of immune recognition, regulation of cell apoptosis, regulation of cell cycle, etc. were selected as the candidate target genes. The minimum free energy of hybridization between miRNA and target gene mRNA was calculated via software RNAhybrid^3^.

### Construction of Lentiviral Expression Vectors and Transfection of HEK293T Cells

To verify the miRNA regulation on target gene expression by binding to its target site in 3′-UTR of mRNA, the lentiviral expression vectors for expression of the target gene and the miRNAs were constructed respectively and transfected into HEK293T cells. The reporter gene *mCherry* sequence (731 bp) was obtained from the plasmid pmCherry-N1. The 570 bp sequence encoding 3′-UTR of the target gene (*BmRan*) mRNA was amplified from the midgut cDNA of the experimental silkworm strain 4008. They were ligated into lentiviral vector pLNHX sequentially to construct the expression vectors pLNHX-mCherry-RanUTR, carrying a recombinant target gene consisting of the reporter gene *mCherry* and the 3′-UTR of *BmRan* mRNA. The *mCherry* gene serves not only as the reporter for successful transfection but also as the substituent target gene for miRNA regulation. At the same time, the miRNA binding sites on the target gene mRNA 3′-UTR were mutated by Mut Express II Fast Mutagenesis Kit V2 (Vazyme Biotech Co., Ltd., China) to construct the expression vector pLNHX-mCherry-RanUTR-mut, in which the miRNA binding sites were destroyed. On the other hand, the miRNA precursor sequence was cloned from the BmCPV genome and inserted into the lentiviral vector pLKO.3G (carrying EGFP reporter gene) to construct the expression vectors pLKO.3G-miR-1 and pLKO.3G-miR-3. The lentiviral vector inserts and primer sequences are shown in Table 2.

**TABLE 2:**
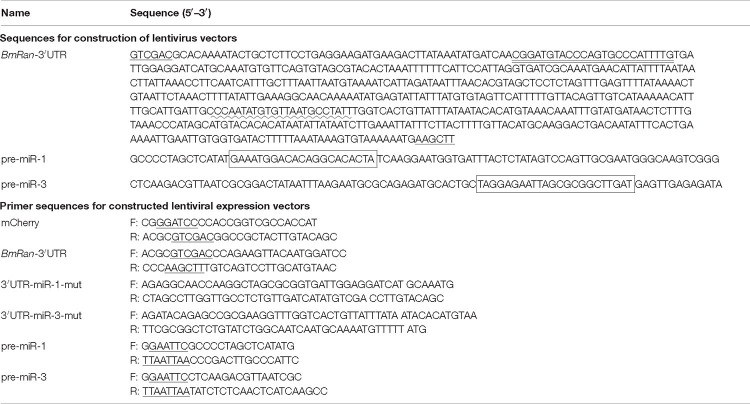
Sequences for construction of lentivirus expression vectors.

*The single-underlined sequence is the restriction site, the double-underlined is the binding site for BmCPV-miR-1, the wavy-underlined is the binding site for BmCPV-miR-3, the boxed sequences are mature miRNAs.*

HEK293T cells were inoculated into a 60 mm cell culture dish in which the cell density should be over 60%, and the transfection was conducted 20 h later. In a 1.5 mL EP tube, 5.3 μg of expression plasmid pLNHX-mCherry-RanUTR or pLNHX-mCherry-RanUTR-mut and 2.7 μg of packaging plasmid pVSV-G were mixed to 125 μL with serum-free DMEM medium (Shanghai Thermo Scientific, China). In another EP tube, 20 μL of transfection reagent (Entranster^TM^-H4000, Beijing Engreen, China) was mixed with 105 μL of serum-free medium and kept at room temperature for 5 min. Then the transfection reagent mixture was mixed thoroughly into the expression plasmid mixture and left to stand for 15 min. For transfection, the medium in the petri dish was removed and the above mixed solution was added to the cells then fresh medium was added up to 5 mL. At 6 h after the transfection, the culture medium was replaced by the fresh medium containing fetal bovine serum (FBS) (Shanghai Thermo Scientific) and the cells were cultured for 24–48 h before the next transfection. When the cells showing red fluorescence accounted for about 80%, they were inoculated into a six-well plate in which the cell density should be over 60%. In a 1.5 mL EP tube, 2 μg of pLKO.3G-miR-1 or pLKO.3G-miR-3, 1.5 μg of packaging plasmid pMD2.G, and 0.5 μg of packaging plasmid pSPAX2 were mixed with serum-free medium to 50 μL. At the same time, 10 μL of transfection reagent (Entranster^TM^-H4000, Beijing Engreen) and 40 μL of serum-free culture medium were mixed in another EP tube and kept at room temperature for 5 min. Then the solution in the two tubes was mixed thoroughly and left to stand for 15 min. The medium in the cell culture was removed, the mixture was added to the cells, and then serum-containing medium was added up to 2 mL. The cells were collected 24 h and 48 h after transfection for RNA extraction and for detection of changes in the expression of reporter gene *mCherry* by qRT-PCR with human β-*actin* as the internal reference.

### BmN Cell Transfection

To verify the miRNA regulation on target gene expression in BmN cells, miRNA mimics were transfected into the BmN cells to detect the changes in expression of the target gene. miRNA mimics and NC (sequences shown in Table 3) were synthesized by Shanghai GenePharma Co., Ltd. BmN cells were inoculated into a six-well plate 16 h before transfection in which the cell density should be over 60%, and they were divided into three groups, i.e., blank control, NC, and mimics, each group was set for three replicates. In a 1.5 mL EP tube, 3.33 μg of mimics or NC was mixed with serum-free TC-100 medium (Sangon Biotech Co., Ltd., Shanghai, China) up to 25 μL. In another tube, 5 μL of transfection reagent (Entranster^TM^-R4000, Beijing Engreen) was mixed with 20 μL of serum-free medium and kept at room temperature for 5 min. The solution in the two tubes was mixed thoroughly and left to stand for 15 min before transfection. For the transfection, the medium in the six-well cell culture plate was removed, the mixture solution was added to the cells, and culture medium was added up to 2.5 mL. Cells were collected at 24, 48, and 72 h after the transfection for RNA extraction and qRT-PCR detection of the changes in target gene expression with *Bm*β-*actin* as internal reference.

**TABLE 3:** Sequences of miRNA mimics, inhibitors, and NC.

Name	Sequence (5′–3′)	
BmCPV-miR-1	Mimic	GAAAUGGACACAGGCACACUA GUGUGCCUGUGUCCAUUUCUU
	Inhibitor	UAGUGUGCCUGUGUCCAUUUC
BmCPV-miR-3	Mimic	UAGGAGAAUUAGCGCGGCUUGAU CAAGCCGCGCUAAUUCUCCUAUU
	Inhibitor	AUCAAGCCGCGCUAAUUCUCCUA

**Negative control (NC)**		AGAAGCUUAGUCGUGUCGGAUGA

### Verification of miRNA Function in Silkworm

To study the regulation of miRNAs on target genes and their influence on virus replication and proliferation in silkworm larvae, the synthesized miRNA mimics, inhibitors (sequence shown in Table 3), and NC were respectively injected into the fifth instar larvae of normal silkworm, 2 μg for each larva. The midgut was dissected at 24, 48, 72, and 96 h after the injection. Total RNA was extracted as described above and the expression level of target gene was detected by qRT-PCR with *Bm*β-*actin* as the internal reference gene.

At the same time, the 5th instar larvae of the silkworm were orally infected with BmCPV, 12 h later, miRNA mimics, inhibitors, and NC were injected respectively into the body cavity of the larvae. At 24, 48, 72, and 96 h after the injection, the midgut tissues were dissected for RNA extraction. Then the replication levels of the second, fifth, and tenth segment of BmCPV genomic RNA were detected respectively by qRT-PCR with *Bm*β-*actin* as internal reference.

### Statistical Analysis Methods

One-way ANOVA analysis in the GraphPad Prism package was used to analyze the experimental data statistically. The results are shown as the mean ± standard error (SE) of three independent treatments. Asterisks denote significant differences as compared with the control group, as indicated by ^∗^*p* ≤ 0.05, ^∗∗^*p* ≤ 0.01, and ^∗∗∗^*p* ≤ 0.001.

## Results

### Stem-Loop PCR Identification of BmCPV-miR-1 and BmCPV-miR-3

**FIGURE 1:**
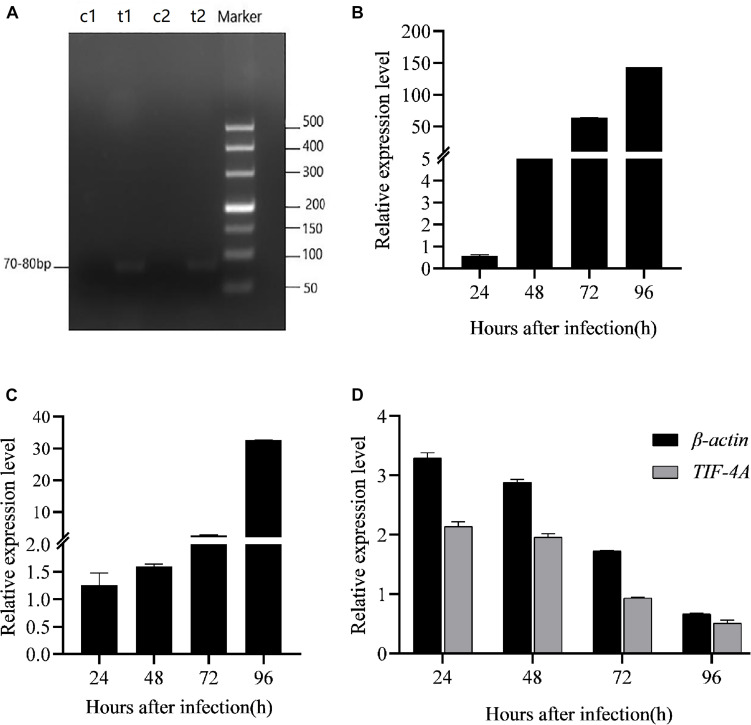
Detection of expression patterns of BmCPV-miRNAs and target gene *BmRan* in the midgut of silkworm larvae infected with BmCPV. **(A)** BmCPV-miR-1 and BmCPV-miR-3 detected by stem-loop RT-PCR. The bands sized about 70–80 bps were the putative miRNAs. c: control; t: BmCPV infected; t1: BmCPV-miR-1; t2: BmCPV-miR-3. **(B)** Expression pattern of BmCPV-miR-1. **(C)** Expression pattern of BmCPV-miR-3. **(D)** Expression pattern of *BmRan* with normalization to different internal reference genes. The expression of BmCPV-miRNAs gradually increased, while the expression of the target gene *BmRan* gradually decreased, as the infection advanced (*n* = 3 replicate samples, each containing five larvae).

In our previous small RNA sequencing data (data deposited in NCBI Sequence Read Archive Database^4^, accession number SRP158739) of the midgut of BmCPV-infected silkworm larvae, we found two miRNA-like small RNAs encoded by the first and third segment of BmCPV genomic RNA, with the sequencing abundances 1375 and 2710, respectively. They were named BmCPV-miR-1 (sequence: GAAAUGGACACAGGCACACUA, located at the 5P arm of its precursor sequence) and BmCPV-miR-3 (sequence: UAGGAGAAUUAGCGCGGCUUGAU, located at the 3P arm of its precursor sequence). With stem-loop RT-PCR detection, obvious bands were detected in midgut tissue of the 5th instar silkworm larvae infected with BmCPV (Figure 1A), with a band size of 70–80 bp respectively, while no bands were detected in midgut tissues of normal larvae, indicating that both BmCPV-miR-1 and BmCPV-miR-3 are derived from BmCPV.

### Target Gene Prediction

miRanda and Targetscan software were used to predict the target genes of both BmCPV-miR-1 and BmCPV-miR-3 against the silkworm genome^5^. As a result, *B. mori* GTP-binding nuclear protein Ran gene (*BmRan*, NCBI accession number: NM_001046809.1) was predicted to be the common target gene of these two miRNAs. The binding sites for the two miRNAs are all located in the 3′-UTR region of the *BmRan* mRNA sequence with an interval of 284 nt. The binding site of BmCPV-miR-1 is located at 11∼34 nt and that of BmCPV-miR-3 is located at 319∼339 nt, both with an individual specific region complementary to the seed sequence of the miRNAs. The binding free energy of the two miRNAs to their respective target sites is lower than −20 kcal/mol (BmCPV-miR-1: −20.8 kcal/mol, BmCPV-miR-3: −22.7 kcal/mol). GTP-binding nuclear protein Ran is a 25 kDa protein and an important component of exportin-5-mediated nucleocytoplasmic transport. It is mainly involved in the transport of small RNA from nucleus to cytoplasm (Bischoff and Ponstingl, 1991). In the process of nuclear export of pre-miRNA, exportin-5 binds pre-miRNA in a Ran-GTP dependent manner. The depletion of Ran protein level leads to a significant reduction in nuclear export of pre-miRNA (Yi et al., 2003; Bohnsack et al., 2004; Lund et al., 2004; Wang et al., 2011). Drosophila exportin-5 can transport pre-miRNA and tRNA (Shibata et al., 2006). However, the mechanism of small RNA transport in the silkworm and its influence factors have not been reported yet. Therefore, the *BmRan* gene of the silkworm was selected in this study to evaluate the function of the two BmCPV-derived miRNAs.

### Expression of *BmRan* Is Inversely Correlated With Both BmCPV-miR-1 and BmCPV-miR-3 Levels in the Larvae Infected With BmCPV

Fifth-instar silkworm larvae were inoculated *per os* with BmCPV, and midgut tissues were collected at 12, 24, 48, 72, and 96 h post-infection. Total RNA was extracted for reverse transcription and for detection of the expressional changes of miRNAs and the target gene. The results indicated that the expression level of BmCPV-miR-1 and BmCPV-miR-3 in the virus-infected midgut gradually increased with the advance of infection (Figures 1B,C), while the expression of the target gene *BmRan* was gradually downregulated as the time advanced after infection (Figure 1D). To ensure the detection objectivity of the target gene expression, two internal reference genes (*Bm*β-*actin* and *BmTIF-4A*) were used to normalize the qRT-PCR detection data. The overall trend in changes of the target gene expression among the different time points after BmCPV infection was the same, although the calculated expression level of the target genes was slightly different at a specific time point as normalized to different reference genes (Figure 1D). It implies that BmCPV-miR-1 and BmCPV-miR-3 might be inversely correlated with the expression of the target gene *BmRan*. It can be speculated that BmCPV-miR-1 and BmCPV-miR-3 have negative regulatory effects on *BmRan*.

### BmCPV-miR-1 and BmCPV-miR-3 Negatively Co-regulate Target Gene Expression by Binding to 3′-UTR of *BmRan* mRNA

The constructed expression vectors pLNHX-mCherry-RanUTR and pLNHX-mCherry-RanUTR-mut, both carrying the recombinant target gene consisting of the 731 bp *mCherry* reporter gene and the 570 bp sequence encoding the *BmRan* mRNA 3′-UTR, were confirmed to be correct by restriction enzyme digestion and sequencing of the inserted fragments. The *mCherry* gene serves here not only as the reporter for successful transfection but also as the substituent target gene for miRNA regulation. On the other hand, the constructed miRNA expression vectors pLKO.3G-miR-1 and pLKO.3G-miR-3 were confirmed by PCR amplification and the specific bands of 87 bp pre-miR-1 and 88 bp pre-miR-3 were detected, then the PCR products were further sequenced as the miRNA precursors of the two miRNAs. These results indicated that the expression vectors were all constructed successfully.

**FIGURE 2:**
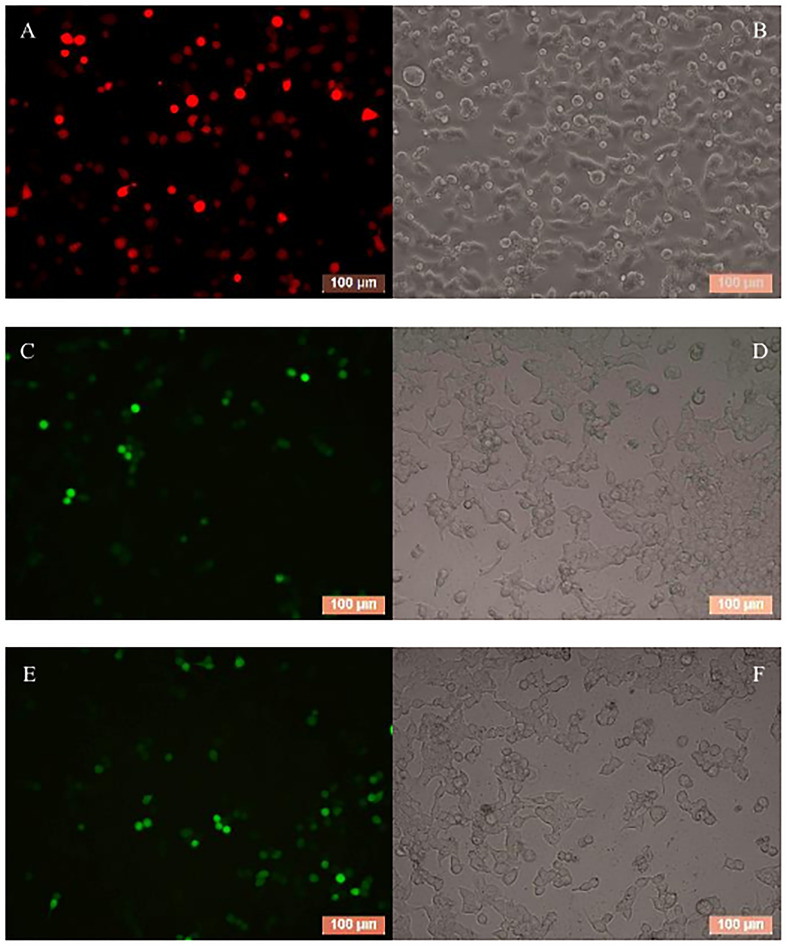
HEK293T cells transfected with the targe gene expression vector pLNHX-mCherry-RanUTR **(A,B)**, the miRNA expression vectors pLKO.3G-miR-1 **(C,D)** and pLKO.3G-miR-3 **(E,F)** respectively. **(A,C,E)** Observed under fluorescent microscope (**A**, under green light; **C,E**, under red light); **(B,D,F)** Observed under optical microscope. Cells showing red fluorescence indicate successful transfection and transfection efficiency of target gene expression vector **(A)**; cells showing green fluorescence indicate the successful transfection and transfection efficiency of miRNA expression vectors **(C,E)**.

**FIGURE 3:**
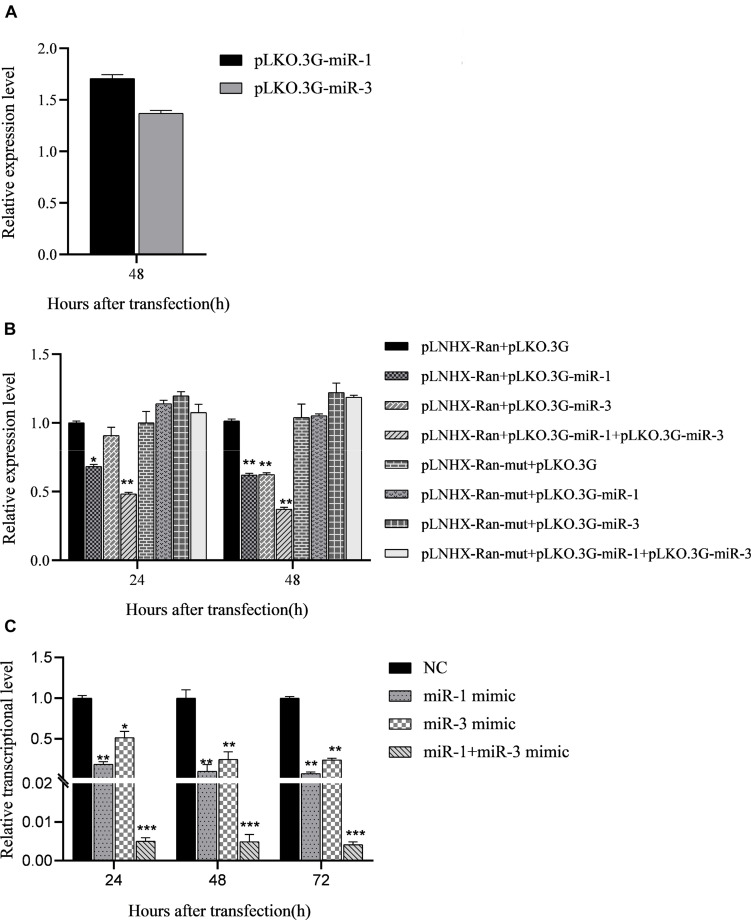
Regulation of BmCPV-miRNAs on the target gene in HEK293T cells and in BmN cells. **(A)** BmCPV-miRNAs were expressed in the transfected HEK293T cells. **(B)** BmCPV-miRNAs downregulate the expression of the substituent target gene *mCherry* in HEK293T cells. pLNHX-Ran: pLNHX-mCherry-RanUTR; pLNHX-Ran-mut: pLNHX-mCherry-RanUTR-mut. **(C)** Regulation of BmCPV-miRNA mimics on expression of target gene *BmRan* in BmN cells (*n* = 3, *p < 0.05, **p < 0.01, ***p < 0.001).

**FIGURE 4:**
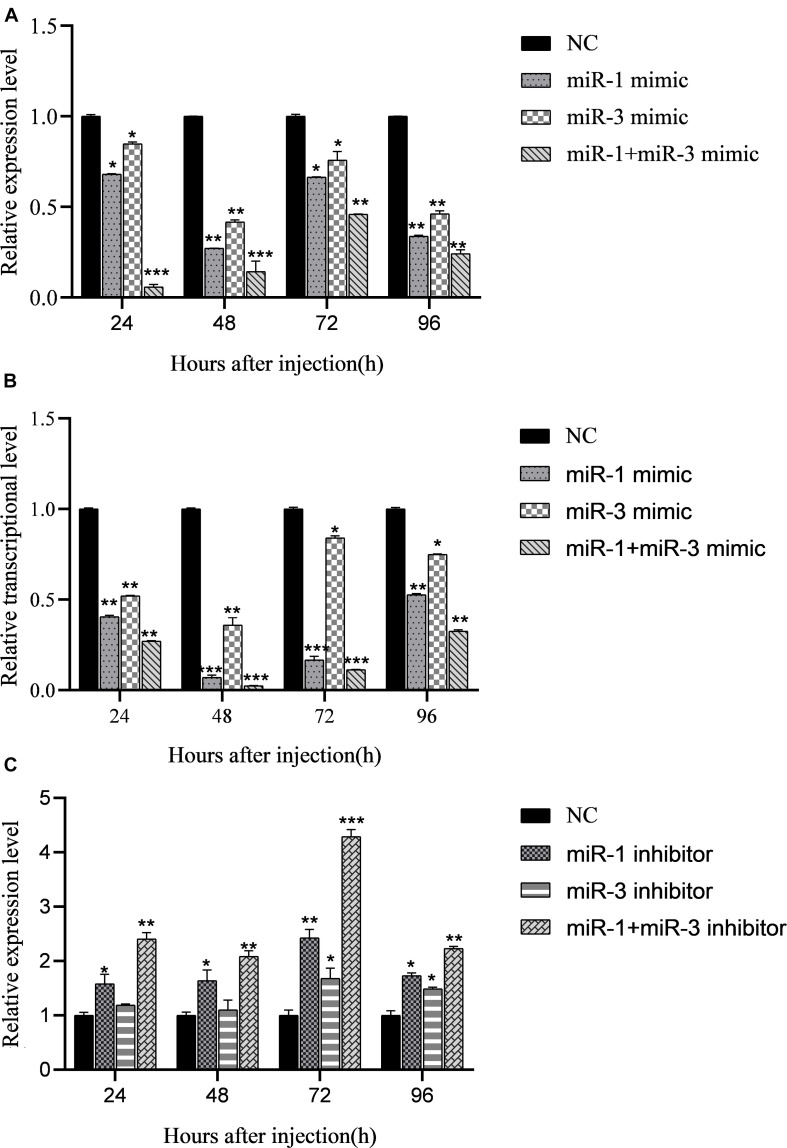
Regulation of miRNA mimics on expression of target gene *BmRan* in midgut of silkworm larvae. Result shows that BmCPV-miRNAs downregulate the expression of *BmRan in vivo* in the silkworm. **(A)** In midgut of normal silkworm larvae. **(B)** In midgut of silkworm larvae infected with BmCPV. **(C)** Regulation of BmCPV-miRNA inhibitors on expression of *BmRan* in midgut of silkworm larvae infected with BmCPV (*n* = 3 replicate samples, each containing five larvae, *p < 0.05, **p < 0.01, ***p < 0.001).

To evaluate the effect of BmCPV-miR-1 and BmCPV-miR-3 bound to the binding sites in 3′-UTR on the expression of the *mCherry* gene (that served as a substituent target gene), HEK293T cells were transfected successively with the above constructed lentiviral expression vectors for mCherry, BmCPV-miR-1, and BmCPV-miR-3. HEK293T cells were firstly co-transfected with the recombinant plasmid pLNHX-mCherry-RanUTR or pLNHX-mCherry-RanUTR-mut and packaging plasmid pVSV-G. After 48 h, about 80% of cells showed red fluorescence (Figures 2A,B), indicating that the recombinant expression vector was successfully transfected into the cells and the red fluorescent protein was stably expressed. Then, the HEK293T cells were further transfected with the plasmid pLKO.3G-miR-1 and pLKO.3G-miR-3 respectively, meanwhile another group of cells transfected with the pLKO.3G plasmid were used as NC. The transfection efficiency was about 60% (Figures 2C–F) at 20 h post-transfection based on the number of cells showing green fluorescence. Then, cells were collected at 24 and 48 h after transfection of the miRNA expression vectors, and the expression of the miRNAs and the red fluorescent protein gene was quantitatively detected. The expression level of both the miRNAs BmCPV-miR-1 and BmCPV-miR-3 gradually increased with the progression of time after transfection of the miRNA expression vectors as detected by qRT-PCR, indicating that the miRNA expression vectors were not only successfully transfected into HEK293T cells, but the miRNAs were also expressed (Figure 3A). At the same time, the expression of the *mCherry* gene in the experimental groups transfected with the miRNA expression vectors decreased (Figure 3B), furthermore the downregulated expression of the *mCherry* gene in the cells co-transfected with both pLKO.3G-miR-1 and pLKO.3G-miR-3 was more significant than that in the cells transfected with only pLKO.3G-miR-1 or pLKO.3G-miR-3 (Figure 4B). While, the expression level of the *mCherry* gene in the NC cells transfected with pLKO.3G was stable (Figure 3B). However, neither pLKO.3G-miR-1 nor pLKO.3G-miR-3 could downregulate the expression of the *mCherry* gene after mutation of the miRNA binding sites on 3′-UTR (Figure 3B). These results implied that both pLKO.3G-miR-1 and pLKO.3G-miR-3 could inhibit the expression of the target gene by binding to 3′-UTR of mRNA, and their cooperative action could enhance the repression of the target gene expression.

### BmCPV-miR-1 and BmCPV-miR-3 Mimics Negatively Regulate *BmRan* Expression in BmN Cells

In order to validate the regulation effect of the two BmCPV-miRNAs on target gene *BmRan*, BmN cells were transfected respectively with BmCPV-miR-1 and BmCPV-miR-3 mimics or NC. Cells were collected at 24, 48, and 72 h after transfection, and the expression changes of the target gene were detected by qRT-PCR. The quantitative detection results (Figure 3C) showed that compared with the cells transfected with NC, the expression of the target gene *BmRan* was downregulated in the cells transfected with the BmCPV-miR-1 mimics or BmCPV-miR-3 mimics. At the same time, the expression of the target gene *BmRan* was downregulated more significantly in the cells co-transfected with both the two miRNA mimics (Figure 3C). It indicated that the mimics of BmCPV-miR-1 and BmCPV-miR-3 could effectively repress the expression of the *BmRan* gene, and furthermore the repression of the expression was enhanced when the mimics of the two miRNAs acted cooperatively. This is consistent with the regulatory effect of BmCPV-miRNAs on the target gene verified by lentiviral expression vectors in HEK293T cells. These results implied the negative regulation of both BmCPV-miR-1 and BmCPV-miR-3 on expression of the target gene *BmRan*.

### BmCPV-miR-1 and BmCPV-miR-3 Downregulate *BmRan* Expression in the Midgut of Silkworm Larvae

To verify the regulatory effects of BmCPV-miR-1 and BmCPV-miR-3 on the expression of the target gene *in vivo* in the silkworm, the mimics of BmCPV-miR-1 and BmCPV-miR-3 were injected into the normal fifth instar larvae separately or jointly to detect the expressional changes of *BmRan*. The larvae injected with NC were used as NC. The results showed that the expression level of the target gene *BmRan* in the midgut of larvae injected with mimics was lower than that in the NC injection group at all the time points of 24, 48, 72, and 96 h after the injection (Figure 4A), and further lower in the larvae injected with both the two miRNA mimics. The results indicated that BmCPV-miR-1 and BmCPV-miR-3 could also inhibit the expression of the target gene *BmRan* in silkworm larvae and have a co-operativity in the repression.

At the same time, the mimics and the inhibitors of BmCPV-miR-1 and BmCPV-miR-3 and the NC were injected into the fifth instar larvae infected with BmCPV to further validate the expression changes of the *BmRan* gene. The results showed that the expression level of the *BmRan* gene in the mimic injection groups was also lower than that in the NC group, with the largest decrease of 40.9 times at 48 h (Figure 4B), while the expression level in the inhibitor injection groups was higher than that in the NC group (Figure 4C) with the highest increase of 4.28 times at 72 h. Furthermore, in the larvae injected with both the two miRNA mimics, the repression of *BmRan* expression was more significantly enhanced, while inhibition of both the miRNAs by injecting both miRNA inhibitors resulted in a much higher *BmRan* expression level. These implied that increasing the level of the BmCPV-miRNAs could inhibit the expression of the *BmRan* gene, while inhibiting the effect of the miRNAs could effectively increase the expression level of the target gene.

### Effect of the BmCPV-miRNAs on BmCPV Replication in the Midgut of Silkworm Larvae

**FIGURE 5:**
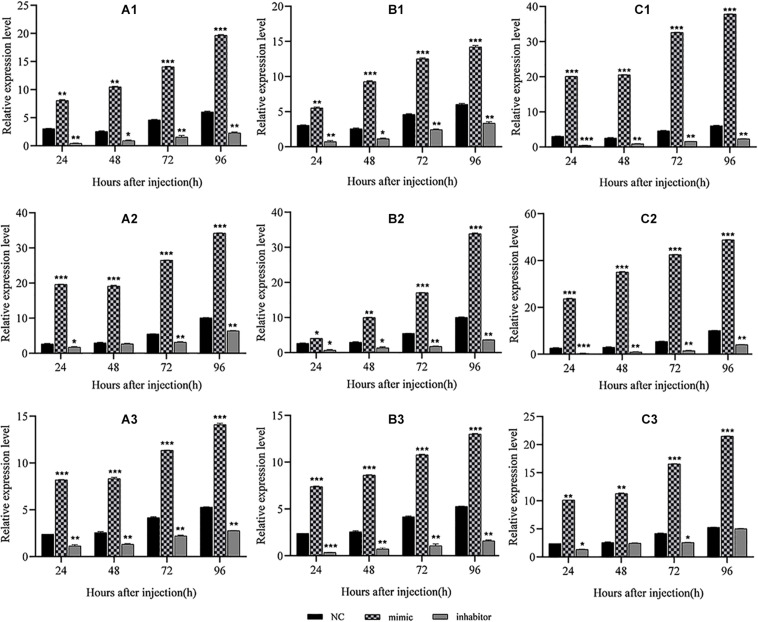
Regulation of miRNA mimics and inhibitors on replication of the BmCPV genome in midgut of silkworm larvae. Result shows that the BmCPV-miRNAs could enhance the replication of BmCPV genomic RNAs. **(A1)** BmCPV-miR-1 to S2; **(B1)** BmCPV-miR-3 to S2; **(C1)** BmCPV-miR-1 + BmCPV-miR-3 to S2; **(A2)** BmCPV-miR-1 to S5; **(B2)** BmCPV-miR-3 to S5; **(C2)** BmCPV-miR-1 + BmCPV-miR-3 to S5; **(A3)** BmCPV-miR-1 to S10; **(B3)** BmCPV-miR-3 to S10; **(C3)** BmCPV-miR-1 + BmCPV-miR-3 to S10. S2, S5, and S10 represent the 2nd, 5th, and 10th segment of BmCPV genomic RNA, respectively (*n* = 3 replicate samples, each containing five larvae, *p < 0.05, **p < 0.01, ***p < 0.001).

The above results verified the negative regulation of the two BmCPV-miRNAs, BmCPV-miR-1 and BmCPV-miR-3, on the target gene *BmRan* both in BmN cells and *in vivo* in the silkworm body. Whether the regulation of BmCPV-miR-1 and BmCPV-miR-3 on the *BmRan* gene affects the replication of the BmCPV genome was also evaluated by injecting the miRNA mimics, inhibitors, and NC into the fifth instar larvae infected with BmCPV and detecting the replication of the second (S2), fifth (S5), and tenth (S10) segment of BmCPV genomic RNA at the time points of 24, 48, 72, and 96 h post-injection. The results showed that the replication levels of the three RNA segments of the viral genome exhibited the same trend, and they all gradually increased with advanced time (Figure 5), which indicated viral replication. While, compared with the NC group, the replication level of the three RNA segments in the silkworm larvae injected with the miRNA mimics increased much faster (Figures 5A1,B1,A2,B2,A3,B3). However, the replication level of the three RNA segments in the larvae injected with inhibitors was lower than that in the NC group (Figures 5A1,B1,A2,B2,A3,B3). Furthermore, increasing the level of both the miRNAs by injecting the two mimics promoted viral replication more significantly than by any single miRNA, while inhibition of both the two miRNAs resulted in further lower replication than inhibition of any single miRNA (Figures 5C1–C3). This indicated that the replication of BmCPV was enhanced by increasing the level of miRNAs, but repressed by a decrease of the miRNA level. Therefore, it can be speculated that BmCPV-miR-1 and BmCPV-miR-3 could create a favorable intracellular environment and thus promote virus replication by inhibiting the expression of the target gene *BmRan*.

## Discussion

Virus-encoded miRNA plays an important role in the process of virus-host interaction. Its small molecules, non-antigenicity, and target specificity make it a potential strategy for the virus to counter host defense mechanisms. Most of the presently reported viral miRNAs are encoded by DNA viruses, but some RNA viruses can also encode functional miRNAs (Swaminathan et al., 2013; Fani et al., 2018), such as Ebola virus (EBOV) (Liang et al., 2014; Chen et al., 2016; Qiu et al., 2018), hepatitis A virus (HAV) (Shi et al., 2014), bovine leukemia virus (BLV) (Rosewick et al., 2013), and Marek’s disease virus (MDV) (Zhao et al., 2011), etc. Studies have shown that one miRNA can target multiple genes, and a target gene can also be regulated by several miRNAs. Generally, miRNA mainly binds to the 3′-UTR of mRNA to repress the target gene translation, and co-operativity between two or more miRNA-binding sites can enhance repression of the mRNA translation (Saetrom et al., 2007; Fang and Rajewsky, 2011; Trobaugh et al., 2014; Liu et al., 2015). On the other hand, some miRNAs bind to the 5′-UTR of mRNA and upregulate the expression of target genes (Jopling et al., 2005; Ørom et al., 2008; Helwak et al., 2013; Hussain et al., 2013). In addition, some miRNAs can also bind to the CDS region and downregulate the expression of the target gene (Hausser et al., 2013; Pan et al., 2017).

*Bombyx mori* cypovirus has a larger and double-stranded RNA genome, which makes the virus potentially able to encode functional miRNAs, and the miRNAs encoded by the virus might play an important role in the virus-host interaction and in virus replication. In the present study, two miRNAs namely BmCPV-miR-1 and BmCPV-miR-3, which are encoded by the first and third segment of BmCPV genomic RNA, respectively, and their regulatory effects on target genes and then on virus replication and proliferation were studied. BmCPV-miR-1 and BmCPV-miR-3 share a common target gene, *B. mori* GTP-binding nuclear protein Ran (*BmRan*), and their binding sites on *BmRan* mRNA are all located in the 3′-UTR region. qPCR results showed that the expression levels of BmCPV-miR-1 and BmCPV-miR-3 in the midgut of virus-infected larvae gradually increased with the progression of infection, while the expression level of the target gene *BmRan* gradually decreased, indicating that both the two miRNAs were negatively correlated with the expression of target gene *BmRan*. Many virally encoded miRNAs were reported to downregulate the expression of host target genes via binding to the 3′-UTR of their mRNA (Stern-Ginossar et al., 2007; Singh et al., 2012; Skalsky et al., 2012; Fani et al., 2018). Therefore, our results implied that BmCPV-miR-1 and BmCPV-miR-3 might downregulate the expression of the target gene *BmRan*.

At present, the regulation of miRNAs on target genes is mostly verified using the dual luciferase reporter system, but both miRNA and the target gene can only be expressed transiently. However, the lentivirus expression system has the characteristics of high transfection efficiency and expression stability. Therefore, we employed the lentivirus expression vectors to express the miRNAs and the target gene respectively, and to evaluate the interaction between the BmCPV-encoded miRNAs and their shared target gene. In the system, the *mCherry* gene and the cDNA sequence encoding 3′-UTR of *BmRan* mRNA were combined into an expression vector, in which the *mCherry* gene served as both the reporter gene for successful transfection and the substituent target gene for the miRNAs. The results showed that both BmCPV-miR-1 and BmCPV-miR-3 could downregulate the expression of the target gene, and furthermore they had a co-operativity in the regulation.

At the same time, the negative regulation of BmCPV-miR-1 and BmCPV-miR-3 on the target gene *BmRan* was also confirmed in the cultured BmN cells by transfection of miRNA mimics, and *in vivo* in the silkworm by injecting miRNA mimics into both the normal and BmCPV-infected silkworm larvae. Furthermore, injecting miRNA mimics into the larvae enhanced the replication of the tested second, fifth, and tenth segment of the viral genome RNA. This implied that BmCPV-miR-1 and BmCPV-miR-3 encoded by BmCPV might promote the replication and proliferation of the virus by inhibiting the expression of the target gene *BmRan*.

In plants and invertebrates such as insects, host miRNAs serve as an important antiviral mechanism and degrade viral RNA into siRNAs, which then bind to the virus genome to inhibit viral replication (Ding and Voinnet, 2007; Carl et al., 2013; Bernier and Sagan, 2018). The biological generation of host miRNAs includes the transcription of pri-miRNA containing a stem-loop structure (Lagos-Quintana et al., 2003), which is then digested into pre-miRNA containing a hairpin structure by the Drosha enzyme in the nucleus. Then the pre-miRNA is transported from the nucleus to the cytoplasm, where it is cleaved into mature miRNA by the Dicer enzyme.

**FIGURE 6:**
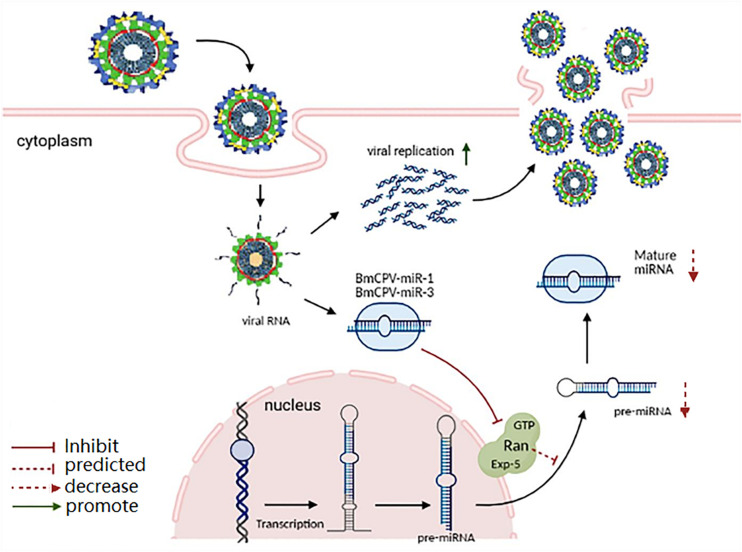
Proposed function mechanism of BmCPV-miR-1 and BmCPV-miR-3 on regulation of the target gene *BmRan* and on replication of BmCPV. BmCPV-miR-1 and BmCPV-miR-3 negatively regulate the translation of the BmRan protein, thus inhibit the transport of pre-miRNA from the nucleus to the cytoplasm in host cells, thereby the population of host miRNAs decreases, consequently creating a favorable intracellular environment for BmCPV genome replication and virus multiplication is enhanced.

GTP-binding nuclear protein Ran is a 25 kDa transporter and serves as an important part of the exportin-5-mediated nucleocytoplasmic transport. It plays an important role in the transport of various non-coding RNAs and proteins from the nucleus to the cytoplasm, and mainly participates in the transport of small RNAs (Bischoff and Ponstingl, 1991). The exportin-5 of Drosophila can transport pre-miRNA and tRNA (Shibata et al., 2006). Research has shown that the nuclear export protein exportin-5 (Exp5) binds specifically to pre-miRNA in a Ran-GTP dependent manner (Yi et al., 2003; Bohnsack et al., 2004; Lund et al., 2004). Then, the pre-miRNA/Exp5/Ran-GTP complex migrates to cytoplasm, where the hydrolysis of Ran-GTP to Ran-GDP induces the release of pre-miRNA. The released pre-miRNA is further processed by the RNase III enzyme called Dicer to release the mature miRNAs (Hutvágner et al., 2001; Ketting et al., 2001). The depletion of Ran results in significant reduction of pre-miRNA export (Yi et al., 2003; Bohnsack et al., 2004; Lund et al., 2004). Other studies have shown that the combination of the pre-miRNA/Exp5/Ran-GTP complex can significantly reduce the efficiency of Dicer cutting pre-miRNA (Kim, 2004; Zeng and Cullen, 2004). In addition, overexpression of the pre-miRNA/Exp5/Ran-GTP complex increased the level of miRNA in the transfected cells, while RNAi-mediated knock-down of the pre-miRNA/Exp5/Ran-GTP complex inhibited the production of mature miRNA. Therefore, the pre-miRNA/Exp5/Ran-GTP complex not only acts as a nuclear export factor for pre-miRNA, but also protects pre-miRNA from degradation, thus promoting the formation of miRNA. Our results indicate that BmCPV-miR-1 and BmCPV-miR-3 may enhance the replication and proliferation of BmCPV by inhibiting the expression of target gene *BmRan*. The probable reason is that BmCPV-miR-1 and BmCPV-miR-3 bind to the 3′-UTR region of *BmRan* mRNA, negatively regulate the translation of the BmRan protein, thus inhibit the transport of pre-miRNA from the nucleus to the cytoplasm in host cells, reduce the population of host miRNAs, and consequently create a favorable intracellular environment for BmCPV genome replication and virus multiplication. Based on this speculation, the function mechanism of BmCPV-miR-1 and BmCPV-miR-3 on regulation of target gene *BmRan* and on replication of BmCPV is proposed (Figure 6). *B. mori* nuclear polyhedrosis virus (BmNPV) is another important virus pathogen of the silkworm, which is a double-stranded DNA virus belonging to *Baculoviridae*. The miRNA bmnpv-miR-1 encoded by BmNPV also represses the expression of *Ran* in the silkworm, leading to the reduction in the host small RNA population, as a consequence, the BmNPV load increases significantly in the infected larvae (Singh et al., 2012). In contrast, blockage of the host miRNA, bmo-miR-8, which targets the immediate-early gene of the virus and whose production was repressed upon bmnpv-miR-1 and Ran dsRNA administration, also resulted in a significant increase in the virus load in the infected silkworm larvae. While inhibition of BmNPV-miR-1 resulted in the significant expression of Ran and the decrease in BmNPV load in the BmNPV-infected larvae (Singh et al., 2012). These results, including those in our present study, provide insights into one of the evasion strategies used by these viruses to counter the host defense for their effective multiplication.

Research on the mechanism of biogenesis of miRNAs by RNA viruses indicated that RNA viruses generate functional miRNAs through non-canonical miRNA biosynthesis pathways. For example, the Drosha enzyme also exists in cytoplasm to cleave pri-miRNA to form pre-miRNA (Shapiro et al., 2012), viruses can encode a protein with the function of the Drosha enzyme to cleave the initial transcripts of miRNA (Kreuze et al., 2005), the tRNase Z in the cytoplasm can cut pri-miRNA into pre-miRNA (Bogerd et al., 2010), and the small stem-loop structure transcripts of the viral genome in the cytoplasm can be directly processed into miRNAs or miRNA-like molecules by the Dicer enzyme (Okamura et al., 2007). Our previous studies identified several miRNAs encoded by BmCPV and have proven that they can regulate the expression of silkworm target genes (Pan et al., 2017; Guo et al., 2020). For example, BmCPV-miR-1 upregulated the expression of another target gene, *B. mori* inhibitor of apoptosis protein (*BmIAP*), by binding to the 5′-UTR of its mRNA and then inhibited the apoptosis of the infected cells, thus facilitating the replication of BmCPV (Pan et al., 2017; Guo et al., 2020). The enhanced replication of the BmCPV genome in the present study should include the contribution from the BmCPV-miR-1 upregulation on *BmIAP*. However, the mechanism with which BmCPV generates the miRNAs requires a further in-depth study in the future.

In summary, the present study revealed the negatively regulatory and co-regulatory function of two BmCPV-encoded putative miRNAs, BmCPV-miR-1 and BmCPV-miR-3, on the host target gene *BmRan*. Furthermore, repression of *BmRan* expression by the two BmCPV-miRNAs enhanced replication of the viral genome. The results might imply one of the strategies employed by the insect virus to modulate miRNA-mediated host antiviral defense by generating miRNAs that inhibits Ran, an important component in miRNA generation.

## Data Availability Statement

The original contributions presented in the study are included in the article/supplementary material, further inquiries can be directed to the corresponding author/s.

## Author Contributions

SL: conceptualization, methodology, investigation, formal analysis, and writing–original draft and editing. YW, ZZ, WW, and YS: methodology, investigation, and formal analysis. ZDZ, MS, and PW: conceptualization, resources, supervision, and writing–original draft. HQ: resources. XG: conceptualization, methodology, funding acquisition, resources, project administration, supervision, and writing–review and editing. All authors contributed to the article and approved the submitted version.

## Conflict of Interest

The authors declare that the research was conducted in the absence of any commercial or financial relationships that could be construed as a potential conflict of interest.

## Publisher’s Note

All claims expressed in this article are solely those of the authors and do not necessarily represent those of their affiliated organizations, or those of the publisher, the editors and the reviewers. Any product that may be evaluated in this article, or claim that may be made by its manufacturer, is not guaranteed or endorsed by the publisher.

## Footnotes

1
http://www.mirbase.org/

2
http://www.targetscan.org/vert_71/

3
https://bibiserv.cebitec.uni-bielefeld.de/rnahybrid

4
http://www.ncbi.nlm.nih.gov/sra/

5
https://www.ncbi.nlm.nih.gov/genome/?term=silkworm

## References

[B1] Bernier A. Sagan S. M. (2018). The diverse roles of microRNAs at the host-virus interface. *Viruses* 10:440. 10.3390/v10080440 30126238 PMC6116274

[B2] Bischoff F. R. Ponstingl H. (1991). Catalysis of guanine nucleotide exchange on Ran by the mitotic regulator RCC1. *Nature* 354 80–82. 1944575 10.1038/354080a0

[B3] Bogerd H. P. Karnowski H. W. Cai X. Shin J. Pohlers M. Cullen B. R. (2010). A mammalian herpesvirus uses noncanonical expression and processing mechanisms to generate viral MicroRNAs. *Mol. Cell* 37 135–142. 10.1016/j.molcel.2009.12.016 20129062 PMC2818755

[B4] Bohnsack M. T. Czaplinski K. Gorlich D. (2004). Exportin 5 is a RanGTP-dependent dsRNA-binding protein that mediates nuclear export of pre-miRNAs. *RNA* 10 185–191. 10.1261/rna.5167604 14730017 PMC1370530

[B5] Cao G. Meng X. Xue R. Zhu Y. Zhang X. Pan Z. (2012). Characterization of the complete genome segments from BmCPV-SZ, a novel *Bombyx mori* cypovirus 1 isolate. *Can. J. Microbiol.* 58 872–883. 10.1139/w2012-064 22712678

[B6] Carl J. W. Jr. Trgovcich J. Hannenhalli S. (2013). Widespread evidence of viral miRNAs targeting host pathways. *BMC Bioinformatics* 14 (Suppl. 2): S3. 10.1186/1471-2105-14-S2-S3 23369080 PMC3549839

[B7] Chang S. Chen W. Yang J. (2009). Another formula for calculating the gene change rate in real-time RT-PCR. *Mol. Biol. Rep.* 36 2165–2168. 10.1007/s11033-008-9430-1 19109763

[B8] Chen C. Ridzon D. A. Broomer A. J. Zhou Z. Lee D. H. Nguyen J. T. (2005). Real-time quantification of microRNAs by stem-loop RT-PCR. *Nucleic Acids Res.* 33:e179. 10.1093/nar/gni178 16314309 PMC1292995

[B9] Chen Z. Liang H. Chen X. Ke Y. Zhou Z. Yang M. (2016). An Ebola virus-encoded microRNA-like fragment serves as a biomarker for early diagnosis of Ebola virus disease. *Cell Res.* 26 380–383. 10.1038/cr.2016.21 26902287 PMC4783470

[B10] Ding S. W. Voinnet O. (2007). Antiviral immunity directed by small RNAs. *Cell* 130 413–426. 10.1016/j.cell.2007.07.039 17693253 PMC2703654

[B11] Fang Z. Rajewsky N. (2011). The impact of miRNA target sites in coding sequences and in 3′UTRs. *PLoS One* 6:e18067. 10.1371/journal.pone.0018067 21445367 PMC3062573

[B12] Fani M. Zandi M. Rezayi M. Khodadad N. Langari H. Amiri I. (2018). The Role of microRNAs in the viral infections. *Curr. Pharm. Des.* 24 4659–4667. 10.2174/1381612825666190110161034 30636585

[B13] Filipowicz W. Bhattacharyya S. N. Sonenberg N. (2008). Mechanisms of post-transcriptional regulation by microRNAs: Are the answers in sight?. *Nat. Rev. Genet.* 9 102–114. 10.1038/nrg2290 18197166

[B14] Gottwein E. Mukherjee N. Sachse C. Frenzel C. Majoros W. H. Chi J. T. (2007). A viral microRNA functions as an orthologue of cellular miR-155. *Nature* 450 1096–1099. 10.1038/nature05992 18075594 PMC2614920

[B15] Grimson A. Farh K. K. Johnston W. K. Garrett-Engele P. Lim L. P. Bartel D. P. (2007). MicroRNA targeting specificity in mammals: determinants beyond seed pairing. *Mol. Cell* 27 91–105. 10.1016/j.molcel.2007.06.017 17612493 PMC3800283

[B16] Guo J. Y. Wang Y. S. Chen T. Jiang X. X. Wu P. Geng T. (2020). Functional analysis of a miRNA-like small RNA derived from *Bombyx mori* cytoplasmic polyhedrosis virus. *Insect Sci.* 27 449–462. 10.1111/1744-7917.12671 30869181

[B17] Hagiwara K. Rao S. Scott S. W. Carner G. R. (2002). Nucleotide sequences of segments 1, 3 and 4 of the genome of *Bombyx mori* cypovirus 1 encoding putative capsid proteins VP1, VP3 and VP4, respectively. *J. Gen. Virol.* 83 1477–1482. 10.1099/0022-1317-83-6-1477 12029163

[B18] Hausser J. Syed A. P. Bilen B. Zavolan M. (2013). Analysis of CDS-located miRNA target sites suggests that they can effectively inhibit translation. *Genome Res.* 23 604–615. 10.1101/gr.139758.112 23335364 PMC3613578

[B19] Heiss B. L. Maximova O. A. Thach D. C. Speicher J. M. Pletnev A. G. (2012). MicroRNA targeting of neurotropic flavivirus: effective control of virus escape and reversion to neurovirulent phenotype. *J. Virol.* 86 5647–5659. 10.1128/jvi.07125-11 22419812 PMC3347253

[B20] Helwak A. Kudla G. Dudnakova T. Tollervey D. (2013). Mapping the human miRNA interactome by CLASH reveals frequent noncanonical binding. *Cell* 153 654–665. 10.1016/j.cell.2013.03.043 23622248 PMC3650559

[B21] Hu X. Chen F. Zhu L. Yu L. Zhu M. Liang Z. (2019). *Bombyx mori* cypovirus encoded small peptide inhibits viral multiplication. *Dev. Comp. Immunol.* 96 51–57. 10.1016/j.dci.2019.02.017 30822453

[B22] Hussain M. Walker T. O’Neill S. L. Asgari S. (2013). Blood meal induced microRNA regulates development and immune associated genes in the Dengue mosquito vector, *Aedes aegypti*. *Insect Biochem. Mol. Biol.* 43 146–152. 10.1016/j.ibmb.2012.11.005 23202267

[B23] Hutvágner G. McLachlan J. Pasquinelli A. E. Bálint E. Tuschl T. Zamore P. D. (2001). A cellular function for the RNA-interference enzyme Dicer in the maturation of the let-7 small temporal RNA. *Science* 293 834–838. 10.1126/science.1062961 11452083

[B24] Jiang L. (2021). Insights into the antiviral pathways of the silkworm *Bombyx mori*. *Front. Immunol.* 12:639092. 10.3389/fimmu.2021.639092 33643323 PMC7904692

[B25] Jopling C. L. Yi M. Lancaster A. M. Lemon S. M. Sarnow P. (2005). Modulation of hepatitis C virus RNA abundance by a liver-specific MicroRNA. *Science* 309 1577–1581. 10.1126/science.1113329 16141076

[B26] Ketting R. F. Fischer S. E. Bernstein E. Sijen T. Hannon G. J. Plasterk R. H. (2001). Dicer functions in RNA interference and in synthesis of small RNA involved in developmental timing in C. elegans. *Genes Dev.* 15 2654–2659. 10.1101/gad.927801 11641272 PMC312808

[B27] Kim V. N. (2004). MicroRNA precursors in motion: exportin-5 mediates their nuclear export. *Trends Cell Biol.* 14 156–159. 10.1016/j.tcb.2004.02.006 15134074

[B28] Kreuze J. F. Savenkov E. I. Cuellar W. Li X. Valkonen J. P. (2005). Viral class 1 RNase III involved in suppression of RNA silencing. *J. Virol.* 79 7227–7238. 10.1128/jvi.79.11.7227-7238.2005 15890961 PMC1112141

[B29] Lagos-Quintana M. Rauhut R. Meyer J. Borkhardt A. Tuschl T. (2003). New microRNAs from mouse and human. *RNA* 9 175–179. 12554859 10.1261/rna.2146903 PMC1370382

[B30] Liang H. Zhou Z. Zhang S. Zen K. Chen X. Zhang C. (2014). Identification of Ebola virus microRNAs and their putative pathological function. *Sci. China Life Sci.* 57 973–981. 10.1007/s11427-014-4759-2 25266153

[B31] Liu G. Zhang R. Xu J. Wu C. I. Lu X. (2015). Functional conservation of both CDS- and 3′-UTR-located microRNA binding sites between species. *Mol. Biol. Evol.* 32 623–628. 10.1093/molbev/msu323 25414126

[B32] Lund E. Güttinger S. Calado A. Dahlberg J. E. Kutay U. (2004). Nuclear export of microRNA precursors. *Science* 303 95–98. 10.1126/science.1090599 14631048

[B33] Okamura K. Hagen J. W. Duan H. Tyler D. M. Lai E. C. (2007). The mirtron pathway generates microRNA-class regulatory RNAs in *Drosophila*. *Cell* 130 89–100. 10.1016/j.cell.2007.06.028 17599402 PMC2729315

[B34] Ørom U. A. Nielsen F. C. Lund A. H. (2008). MicroRNA-10a binds the 5′UTR of ribosomal protein mRNAs and enhances their translation. *Mol. Cell* 30 460–471. 10.1016/j.molcel.2008.05.001 18498749

[B35] Pan Z. H. Wu P. Gao K. Hou C. X. Qin G. X. Geng T. (2017). Identification and characterization of two putative microRNAs encoded by *Bombyx mori* cypovirus. *Virus Res.* 233 86–94. 10.1016/j.virusres.2017.03.009 28286035

[B36] Qiu G. H. Weng Z. H. Hu P. P. Duan W. J. Xie B. P. Sun B. (2018). Synchronous detection of ebolavirus conserved RNA sequences and ebolavirus-encoded miRNA-like fragment based on a zwitterionic copper (II) metal-organic framework. *Talanta* 180 396–402. 10.1016/j.talanta.2017.12.045 29332829

[B37] Rosewick N. Momont M. Durkin K. Takeda H. Caiment F. Cleuter Y. (2013). Deep sequencing reveals abundant noncanonical retroviral microRNAs in B-cell leukemia/lymphoma. *Proc. Natl. Acad. Sci. U.S.A.* 110 2306–2311. 10.1073/pnas.1213842110 23345446 PMC3568357

[B38] Saetrom P. Heale B. S. Snøve O. Jr. Aagaard L. Alluin J. Rossi J. J. (2007). Distance constraints between microRNA target sites dictate efficacy and cooperativity. *Nucleic Acids Res.* 35 2333–2342. 10.1093/nar/gkm133 17389647 PMC1874663

[B39] Shapiro J. S. Langlois R. A. Pham A. M. Tenoever B. R. (2012). Evidence for a cytoplasmic microprocessor of pri-miRNAs. *RNA* 18 1338–1346. 10.1261/rna.032268.112 22635403 PMC3383965

[B40] Shi J. Duan Z. Sun J. Wu M. Wang B. Zhang J. (2014). Identification and validation of a novel microRNA-like molecule derived from a cytoplasmic RNA virus antigenome by bioinformatics and experimental approaches. *Virol. J.* 11:121. 10.1186/1743-422x-11-121 24981144 PMC4087238

[B41] Shibata S. Sasaki M. Miki T. Shimamoto A. Furuichi Y. Katahira J. (2006). Exportin-5 orthologues are functionally divergent among species. *Nucleic Acids Res.* 34 4711–4721. 10.1093/nar/gkl663 16963774 PMC1635293

[B42] Singh C. P. Singh J. Nagaraju J. (2012). A baculovirus-encoded MicroRNA (miRNA) suppresses its host miRNA biogenesis by regulating the exportin-5 cofactor Ran. *J. Virol.* 86 7867–7879. 10.1128/jvi.00064-12 22593162 PMC3421685

[B43] Skalsky R. L. Corcoran D. L. Gottwein E. Frank C. L. Kang D. Hafner M. (2012). The viral and cellular microRNA targetome in lymphoblastoid cell lines. *PLoS Pathog.* 8:e1002484. 10.1371/journal.ppat.1002484 22291592 PMC3266933

[B44] Stern-Ginossar N. Elefant N. Zimmermann A. Wolf D. G. Saleh N. Biton M. (2007). Host immune system gene targeting by a viral miRNA. *Science* 317 376–381. 10.1126/science.1140956 17641203 PMC4283197

[B45] Swaminathan G. Martin-Garcia J. Navas-Martin S. (2013). RNA viruses and microRNAs: challenging discoveries for the 21st century. *Physiol. Genomics* 45 1035–1048. 10.1152/physiolgenomics.00112.2013 24046280 PMC3841790

[B46] Swevers L. Feng M. Ren F. Sun J. (2020). Antiviral defense against Cypovirus 1 (Reoviridae) infection in the silkworm, *Bombyx mori*. *Arch. Insect Biochem. Physiol.* 103:e21616. 31502703 10.1002/arch.21616

[B47] Trobaugh D. W. Gardner C. L. Sun C. Haddow A. D. Wang E. Chapnik E. (2014). RNA viruses can hijack vertebrate microRNAs to suppress innate immunity. *Nature* 506 245–248. 10.1038/nature12869 24352241 PMC4349380

[B48] Wang X. Xu X. Ma Z. Huo Y. Xiao Z. Li Y. (2011). Dynamic mechanisms for pre-miRNA binding and export by Exportin-5. *RNA* 17 1511–1528. 10.1261/rna.2732611 21712399 PMC3153975

[B49] Yi R. Qin Y. Macara I. G. Cullen B. R. (2003). Exportin-5 mediates the nuclear export of pre-microRNAs and short hairpin RNAs. *Genes Dev.* 17 3011–3016. 10.1101/gad.1158803 14681208 PMC305252

[B50] Zeng Y. Cullen B. R. (2004). Structural requirements for pre-microRNA binding and nuclear export by Exportin 5. *Nucleic Acids Res.* 32 4776–4785. 10.1093/nar/gkh824 15356295 PMC519115

[B51] Zhao Y. Xu H. Yao Y. Smith L. P. Kgosana L. Green J. (2011). Critical role of the virus-encoded microRNA-155 ortholog in the induction of Marek’s disease lymphomas. *PLoS Pathog.* 7:e1001305. 10.1371/journal.ppat.1001305 21383974 PMC3044692

[B52] Zhao Y. Yao Y. Xu H. Lambeth L. Smith L. P. Kgosana L. (2009). A functional MicroRNA-155 ortholog encoded by the oncogenic Marek’s disease virus. *J. Virol.* 83 489–492. 10.1128/jvi.01166-08 18945769 PMC2612317

